# Adaptive evolution of the chrysanthemyl diphosphate synthase gene involved in irregular monoterpene metabolism

**DOI:** 10.1186/1471-2148-12-214

**Published:** 2012-11-08

**Authors:** Ping-Li Liu, Jun-Nan Wan, Yan-Ping Guo, Song Ge, Guang-Yuan Rao

**Affiliations:** 1 College of Life Sciences, Peking University, Beijing, 100871, China; 2 College of Life Sciences, Beijing Normal University, Beijing, 100875, China; 3 State Key Laboratory of Systematic and Evolutionary Botany, Institute of Botany, Chinese Academy of Sciences, Beijing, 100093, China

## Abstract

**Background:**

Chrysanthemyl diphosphate synthase (CDS) is a key enzyme in biosynthetic pathways producing pyrethrins and irregular monoterpenes. These compounds are confined to plants of the tribe Anthemideae of the Asteraceae, and play an important role in defending the plants against herbivorous insects. It has been proposed that the CDS genes arose from duplication of the farnesyl diphosphate synthase (FDS) gene and have different function from *FDS*s. However, the duplication time toward the origin of *CDS* and the evolutionary force behind the functional divergence of the CDS gene are still unknown.

**Results:**

Two duplication events were detected in the evolutionary history of the FDS gene family in the Asteraceae, and the second duplication led to the origin of *CDS*. *CDS* occurred after the divergence of the tribe Mutisieae from other tribes of Asteraceae but before the birth of the Anthemideae tribe. After its origin, CDS accumulated four mutations in sites homologous to the substrate-binding and catalysis sites of FDS. Of these, two sites were involved in the binding of the nucleophilic substrate isopentenyl diphosphate in FDS. Maximum likelihood analyses showed that some sites in CDS were under positive selection and were scattered throughout primary sequences, whereas in the three-dimensional structure model they clustered in the large central cavity.

**Conclusion:**

Positive selection associated with gene duplication played a major role in the evolution of *CDS.*

## Background

Chrysanthemyl diphosphate synthase (CDS) catalyzes the condensation of two molecules of dimethylallyl diphosphate (DMAPP) to form chrysanthemyl diphosphate and is a key enzyme in biosynthetic pathways involving the production of pyrethrins and irregular monoterpenes [1-4]. Irregular monoterpenes are much less common than other isoprenoids and are confined to plants of the tribe Anthemideae in the family Asteraceae [1,2,5]. These secondary metabolites play an important role in their defense against herbivorous insects [1,2,4,6-8].

Many enzymes involved in secondary plant metabolism are encoded by gene families that originated through gene duplications [9-11]. Evidence shows that the CDS genes resulted from duplication of the farnesyl diphosphate synthase (FDS) genes that belong to a small family [2,12-14], with copy numbers ranging from one in grape and two in *Arabidopsis thaliana,* to five in rice [15]. In *Artemisia tridentata* (Asteraceae-Anthemideae), three FDS genes have been identified: *FDS1*, *FDS2* and *CDS* (also known as *FDS5*) [2]. Despite the high sequence similarity of the three genes, divergent functions have been found among them [1,2]. FDS1 and FDS2 catalyze the sequential head-to-tail condensation of two molecules of isopentenyl diphosphate (IPP) with DMAPP to produce farnesyl diphosphate (FPP), and are involved in the biosynthesis of regular sesquiterpenes [2]; whereas CDS catalyzes two molecules of DMAPP to form irregular monoterpenes [1,2]. Moreover, FDS and its products are found in organisms ranging from prokaryotes to eukaryotes [16,17], while the products of CDS are only present in Anthemideae [1,2,5].Thus, *FDS* seems to be an ancient gene of which *CDS* is a derived or modified orthologous copy, and irregular monoterpenes might be products of a pathway arising from those of other isoprenoids.

Gene duplication is prevalent in plant genomes and the duplicated genes face different evolutionary fates, including pseudogenization (nonfunctionalization), retention of the original function, subfunctionalization, and neofunctionalization under the functional view [18-25]. The duplication origin and distinct function of *CDS* raise a number of interesting questions. First, when did the duplication event leading to the origin of *CDS* take place? Because the products of CDS occur exclusively in Anthemideae [1,2,5], it would be expected that the CDS gene occurred at the same time as the origin of this tribe. To date, *CDS* sequences have only been cloned from two species of Anthemideae (*Pyrethrum cinerariifolium* and *A. tridentata*) [1,2] and no information is available as to whether *CDS* occurs in any other members of the Anthemideae and the related relatives. Second, given the fact that CDS uses a new nucleophilic substrate to generate new products, did CDS accumulate mutations in sites homologous to the substrate-binding and catalysis sites of FDS? FDS contains five conserved regions, two of which are DDXXD motifs [1,2,26]. At the three-dimensional structure level, conserved amino-acids in the five regions and the C-terminal of FDS are located in a large central cavity surrounded by 10 α-helices, which have been identified as substrate-binding and catalysis sites [27-31]. Previous studies demonstrated that CDS has a T **→** G substitution in region IV and a D **→** N substitution in the first aspartate in region V [1,2].

Finally, what was the evolutionary force leading to the functional divergence of *CDS*? It is controversial whether the functional divergence of duplicate genes arises from the relaxation of selective constraints or positive selection [19,23,32,33]. Generally, neutral evolution with relaxed selective constraints is treated as the null hypothesis [19,34] and positive selection is invoked if the null hypothesis is rejected. Positive selection has been detected in many genes after their duplication [33,35]. It has been proposed that positive selection promotes the functional divergence of gene family members encoding enzymes involved in secondary metabolism [36,37]. Because CDS carries out the production of irregular monoterpenes that are important secondary metabolites for defense against herbivorous insects in Anthemideae, we investigated whether the functional divergence of CDS was driven by positive selection.

In this study, we reconstructed the phylogeny of the FDS gene family based on cDNA and EST sequences from the main Asteraceae lineages. We detected two rounds of gene duplication during the evolution of the Asteraceae FDS gene family, and inferred the possible time of origin of the CDS gene. Homology modeling and molecular evolutionary analyses showed that two mutations in CDS might be responsible for the fact that CDS does not prefer IPP as the nucleophilic substrate like FDS, and demonstrated that positive selection has played a role in the functional divergence of CDS in Anthemideae.

## Methods

### Amplification of *FDS* homologs from Anthemideae and its relatives

**Figure 1:**
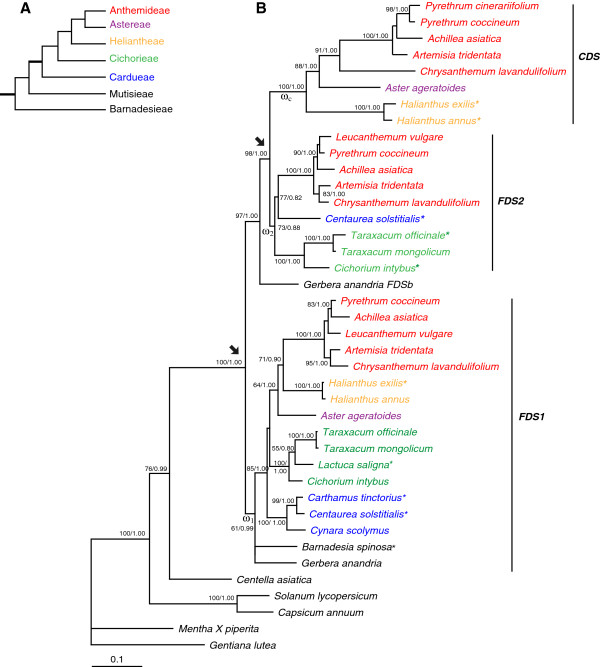
**Phylogenetic relationships of major clades of Asteraceae and phylogeny of the FDS gene family.** (**A**) Phylogenetic relationships of major clades of Asteraceae modified from the Figure 1 of Panero & Funk [41]. Bold branches indicate those most likely for the duplication event in the CDS family. (**B**) Phylogeny of the FDS gene family reconstructed by the ML method. Numbers next to branches are bootstrap percentages from Maximum Likelihood analysis, and posterior probabilities from Bayesian analysis. The accession numbers of each sequence are given in Additional file 2. The arrows indicate the duplication events. The asterisks indicate sequences that were not used in the codon-based analysis because of their incomplete coding regions. The colors indicate the tribes to which the species belong.

Previous studies have well resolved the major clades of Asteraceae and their relationships, with the Anthemideae and Astereae tribes being most closely related [38-41] (Figure 1A). In this study, four species representing four subtribes of Anthemideae (*Pyrethrum coccineum*, *Leucanthemum vulgare*, *Achillea asiatica*, and *Chrysanthemum lavandulifolium*) were sampled. We also sampled one representative species from each of four tribes: *Aster ageratoides* (Astereae), *Helianthus annuus* (Heliantheae), *Taraxacum mongolicum* (Cichorieae), and *Gerbera anandria* (Mutisieae).

We first amplified *FDS* homologs from genomic DNA and then obtained the full-length cDNA using gene-specific primers based on the partial DNA fragments. Genomic DNA was extracted using a Plant Genomic DNA Kit (TianGen Biotech., Beijing, China). Two cycles of polymerase chain reaction (PCR) were conducted to amplify sequences corresponding to conserved regions II through V of *FDS* and *CDS*. In the first round of PCR, the primers CDSII (5^′^-CTTSTMCWTGATGACATRATGGA-3^′^) and CDSVb (5^′^-TGCATTCTTCAATATCTGTTCCMGT-3^′^) were used to amplify *CDS*, while the primers FDSII (5′-CTKGTRCTYGATGAYATYATGGA-3^′^) and FDSVb (5^′^-TKAARKCTTCWATRTCKGTYCCWAT-3^′^) were used to amplify *FDS*. In the second round of PCR, the primers CDSII and CDSVa (5^′^-CRAAAGTGTCGAGATAATCATT-3^′^) were used to amplify *CDS*, and FDSII and FDSVa (5^′^-CAAAACARTCBAGATAATCRTCCT-3^′^) were used to amplify *FDS*. Each amplification reaction (20 μl) contained 1× buffer, 0.5 μM of each primer, 200 μM of each dNTP, and 2.5 U of Taq polymerase, to which 1–1.5 μl of each genomic DNA template was added. The thermocycling program comprised an initial 5 min at 95°C, followed by 35 cycles of 1 min at 95°C, 1 min at 52–58.5°C depending on the DNA template, and 1 min at 72°C, with a final extension step of 5 min at 72°C. The amplification products were gel-purified and cloned into pGEM-T vectors (Promega Corp., Madison, USA). Twenty positive clones were screened using restriction enzyme fragment analysis. All distinct clones with the correct insertion were sequenced and the products were run on an ABI3730 automatic sequencer.

Total RNA from a mixture of leaves and shoots was extracted using a TRIzol kit (Tiangen Biotech Co., Ltd, Beijing, China), and 5^′^ rapid amplification of cDNA ends (5^′^ RACE) was performed using the 5^′^ RACE system (Invitrogen, USA). After performing first-strand cDNA synthesis with gene-specific primers, the original mRNA was removed with RNase H and RNase T1, and a polyC tail was added to the 5^′^-end. Then, two rounds of PCR amplification were performed with nested primers (Additional file 1). For 3^′^ RACE, first-strand cDNA was produced using SuperScript^TM^ II Reverse Transcriptase (Invitrogen, USA). Then, one or two rounds of PCR amplification were carried out with nested primers (Additional file 1). The amplification products (5^′^RACE and 3^′^RACE) were purified, cloned into pGEM-T vectors (Promega Corp., Madison, USA) and sequenced. The sequences were deposited in GenBank (accession numbers in Additional file 2).

### EST database search

A survey of GenBank revealed that more than 1 million expressed sequence tag (EST) sequences were available for species from five tribes of Asteraceae [42]. Most of these sequences were contributed by the Compositae Genome Project. In the present study, libraries with more than 15,000 ESTs were used. For genera such as *Helianthus*, although EST libraries for several species are available, we used only one species to represent each genus. Hence, EST libraries for nine Asteraceae species representing five tribes were downloaded from GenBank. These species were *Helianthus exilis* (Heliantheae), *Cichorium intybus*, *Lactuca saligna* and *Taraxacum officinale* (Cichorieae), *Centaurea solstitialis*, *Carthamus tinctorius* and *Cynara scolymus* (Cardueae), *Gerbera hybrida* (Mutisieae), and *Barnadesia spinosa* (Barnadesieae). A Blast database for each EST library was constructed using the Formatdb program implemented in stand-alone Blast 2.2.13 software [43]. Blast searches for sequences similar to *FDS* in each database were conducted using the TBlastN program with the FDS1 protein sequence of *A. tridentata*[2] as the query. Overlapping ESTs were assembled manually. Detailed information for contigs and singletons (represented by single reads) is listed in Additional file 2.

### Phylogenetic analysis

Phylogenetic analyses were based on the coding sequences. The *FDS2*-like sequence of *Lactuca saligna* was excluded because of a large stretch of missing bases (240 bp) at the N-terminal. Sequences were aligned based on the translated amino-acid sequences using ClustalW in DAMBE [44].

Five additional FDS sequences from species of Asterids were retrieved from NCBI nonredundant sequence databases, with one sequence from a Gentianaceae species as the outgroup (for sequence information, see Additional file 2). Maximum likelihood (ML) analysis implemented in PHYML version 3.0 [45] and Bayesian inference (BI) implemented in MrBayes version 3.1.2 [46] were used to construct phylogenetic trees. The best-fit evolutionary model, GTR + I + G, was selected with the Akaike information criterion using MODELTEST 3.06 [47]. For the ML analysis, the starting tree was obtained with BioNJ, and parameter values were estimated from the data. Branch support was estimated from 1000 bootstrap replicates (BP). In the BI analysis, two independent Markov chain Monte Carlo runs were run simultaneously starting from a random tree for 10 million generations, sampled every 1000 generations. The first 10% of samples were discarded as burn-in, and the remaining trees were used to construct the 50% majority-rule consensus tree.

### Selection test

The codeml program in the PAML 4b package [48] was used to analyze possible positive selection acting on the FDS gene family. To reduce the impact of missing sites, our analyses were limited to FDS genes that contained the full-length coding region. First, branch models allowing the ω ratio (ω = d_N_/d_S_; where d_N_ is the non-synonymous substitution rate and d_S_ is the synonymous substitution rate) to vary among lineages [49] were used to determine whether the selective pressure differed among different lineages. The one ratio model (M0) assumes the same ω for all branches and all sites. The free ratio model (Mf) assumes an independent ω parameter for each branch in the tree. In the phylogenetic analyses, three major clades, *FDS1*, *FDS2*, and *CDS*, were resolved. We assigned ω_1_, ω_2_, and ω_c_ to the lineages ancestral to the *FDS1*, *FDS2*, and *CDS* clades, respectively. The two ratio models (M2a-M2c) assumed one ω ratio for branches of interest and the other ratio, ω_0_, for all other branches; e.g., M2c assumed ω_c_ for the branch ancestral to *CDS* and ω_0_ for all other branches (ω_1_ = ω_2_ = ω_0_). The three ratio models (M3a-M3c) assumed two branches of interest with different ω ratios and all other branches had a ratio of ω_0._ A more complex four ratio model (M4a) assumed four independent ω ratios: one ratio each for the ancestral branches of *FDS1* (ω_1_), *FDS2* (ω_2_), and *CDS* (ω_c_), and one for all other branches (ω_0_). These models were compared using likelihood ratio tests (LRTs) of the log likelihood (InL) to check which model fit the data significantly better.

Because the branch models average the ω ratio over all sites and were unable to detect a positive signal in many cases, site-specific models allowing ω to vary among sites [50,51] were subsequently used to determine whether particular amino-acid residues within *FDS* gene families have been subject to positive selection. In addition to the one ratio model (M0), five site models (M1, M2, M3, M7, and M8) [50,51] were used. The nearly neutral model (M1) assumes two classes of sites: conserved sites under strict constraint (0 < ω < 1) and others under neutral selection (ω = 1). The positive selection model (M2) is an extension of M1 and assumes a third class of positively-selected sites (ω > 1). The discrete model (M3) uses a general discrete distribution with three site classes. The beta model (M7) assumes a beta distribution for the ω ratios over sites, while the beta&ω model (M8) adds another site class to M7, allowing the ω values to exceed 1. Three LRTs of nested models were applied: M0 *versus* M3, M1 *versus* M2, and M7 *versus* M8.

As the branch model showed that the ω value for the branch ancestral to *CDS* was significantly different from that for the other branches, we further used branch-site model A to test for sites that were potentially under positive selection on the branch ancestral to the *CDS* subfamily [52]. The model assumes four classes of sites. The first two have ω_0_ (0 < ω_0_ < 1) and ω_1_ (ω_1_ = 1) along all lineages in the phylogeny, whereas the third and fourth have ω_2_ along the ancestral *CDS* branch, but ω_0_ and ω_1_ along other background branches. The branch-site model A was compared with the nearly neutral model (M1).

### Structure modeling

A homology model was constructed for CDS (I13995) based on the crystal structure of human FDS in complex with zoledronate and isopentenyl diphosphate (Protein Data Bank Accession 2F8Z). The first 50 residues in the N-terminal of CDS were cut off because this portion is removed in the mature CDS protein [1]. The Align 2D structure alignment program (InsightII; Accelrys, San Diego, CA) was used to align the sequences, and the MODELER module of InsightII was used to automatically generate models [53]. To select the best model, all optimized models were evaluated using the Profile-3D program [54]. Molecular graphics were created with PyMOL [55].

## Results

### Characterization of the FDS gene family

In total, we cloned 19 full-length cDNA sequences for the FDS gene family from 8 species of Asteraceae. For *CDS* in *Leucanthemum vulgare* and *FDS2* in *Aster ageratoides,* we were unable to isolate a full-length cDNA despite great attempts using different cDNA templates, primers, PCR programs and annealing temperatures. Alignment of ORF sequences revealed a 2-base insertion in *Helianthus annuus CDS,* which was confirmed by repeated PCR amplification, cloning, and sequencing. This frameshift mutation leads to a premature stop codon, indicative of a nonfunctional pseudogene.

The length of the *cFDS* sequences ranged from 1194 to 1470 bp, with the ORF ranging from 1029 to 1035 bp, encoding proteins of 342 to 344 amino-acids. With the exception of the *A. ageratoides* c*CDS*, which included a large indel in the N-terminal, the *cCDS* sequences varied in length from 1330 to 1430 bp, with the ORF having a length of 1182 to 1197 bp, encoding proteins of 394 to 399 amino-acids. Compared to *FDS*, *CDS* exhibited an ~50-amino acid extension at its N-terminal. The extension sequences of *CDS* from different species were highly variable, being rich in serine and threonine residues (average 22.7% serine, 9.44% threonine) and showing a lack of acidic amino-acids. They were identified as potential chloroplast transit peptides by TargetP Version 1.1 [56]. These peptides shared little similarity with any other database sequence entries based on Blast searches.

Sixteen FDS contigs and singletons were obtained from nine EST libraries, of which thirteen had a length of more than 540 bp. However, there were only three sequences with a full-length coding sequence: the FDS1 genes from *Cichorium intybus*, *Taraxacum officinale* and *Cynara scolymus.*

### Phylogeny of the FDS gene family

Two phylogenetic methods (ML and BI) generated almost the same tree topology and thus only ML tree is shown, with the internal node supports from two methods (Figure 1B). The ML tree clearly showed that all *FDS* sequences from Asteraceae form a well-supported clade. *FDS* sequences from the species of other families fell out of the Asteraceae clade. In this clade, there were three clusters of genes, one consisting of *FDS1* homologs, one *FDS2* homologs and another *CDS* homologs, indicative of two duplication events happened in the evolution of *FDS* gene family of Asteraceae. *CDS* clade was sister to that of *FDS2*, suggesting that the second duplication event led to the origin of *CDS*. All the Anthemideae species (marked by red colour in Figure 1B) had FDS1, FDS2 and CDS genes, whereas only two types of *FDS* homologs were obtained from most species of other tribes. In *Aster ageratoides* of the tribe Astereae and *Helianthus* species of Heliantheae, both *FDS1* and *CDS* homologs were found. In species of Cichorieae and Cardueae, one type of *FDS* copy formed a cluster with the *FDS1* clade and another with the *FDS2* clade. Interestingly, two *FDS* homologs were cloned from *Gerbera anandria* of Mutisieae, the basal tribe of Asteraceae. One of them fell in *FDS1* clade, while another clustered with the *FDS2+CDS* ancestral clade. These findings indicated that the second duplication event giving rise to *FDS2* and *CDS* occurred after the divergence of *G. anandria* and before the origin of the tribe Anthemideae. *Barnadesia spinosa*, a species from the most basal tribe Barnadesieae (Figure 1A), had one FDS homolog clustered with *FDS1,* suggesting that the first duplication event occurred in the common ancestor of Asteraceae.

### Structure modeling

**Figure 2:**
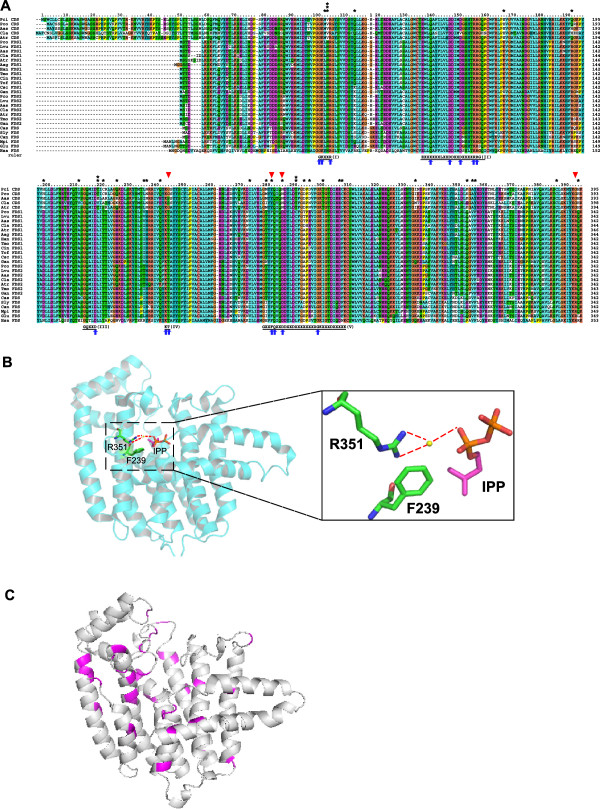
**Alignment of deduced amino-acid sequences and three-dimensional models of FDS and CDS.** (**A**) Multiple sequence alignment of FDS and CDS. The amino-acid positions are numbered relative to CDS in *P. cinerariifolium* (I13995). The consensus sequence highlights the five conserved domains identified in FDS. Sites under positive selection identified by PAML are indicated by asterisks (*sites with p>80%, **sites with p>95%, ***sites with p>99%). Amino-acids involved in the binding and catalysis of substrates [29-31,60] are marked by blue arrows. Red triangles show four sites involved in the binding and catalysis of substrates in FDS and mutated in *CDS*. Species name abbreviations: Aas, *Achillea asiatica*; Atr, *Artemisia tridentata*; Aag, *Aster ageratoides*; Can, *Capsicum annuum*; Cas, *Centella asiatica*; Cin, *Cichorium intybus*; Cla, *Chrysanthemum lavandulifolium*; Csc, *Cynara scolymus*; Glu,*Gentiana lutea*; Gan, *Gerbera anandria*; Han, *Helianthus annuus*; Lvu, *Leucanthemum vulgare*; Hsa, *Homo sapiens* (NP_001129294) (The human sequence is included for alignment since it was used as a template in the homology modeling)*;* Mpi, *Mentha x piperita*; Pci, *Pyrethrum cinerariaefolium*; Pco *Pyrethrum coccineum*; Sly, *Solanum lycopersicum*; Tmo, *Taraxacum mongolicum;* Tof*,Taraxacum officinale*. (**B**) Three-dimensional model of *FDS* (PDB: 2F8Z)*.* R351 and F239 are involved in IPP-binding in FDS. The red dashed lines represent hydrogen bonds. The yellow sphere indicates water. (**C**) Distribution of positively-selected sites in the homology model of CDS. CDS has a structural model very similar to FDS: the arrangement of 10 core helices around a large central cavity. The sites indentified as positively-selected (p>80%) were clustered in the large central cavity (shown in pink).

Previous studies demonstrated that the following sixteen FDS residues are involved in substrate-binding and catalysis: 56G, 57K, 60R, 96Q, 103D, 107D, 112R, 113R, 174D, 200K, 201T, 239F, 240Q, 243D, 257K, and 351R (referred to 2F8Z) (Figure 2A, indicated by blue arrows) [29-31]. Among these sites of FDS, four were mutated in homologous sites of CDS: T201 in FDS **→** G244 (or S244) in CDS; F239 in FDS **→** Y281 in CDS; D243 in FDS **→** N285 in CDS; and R351 in FDS **→** G393 in CDS (Figure 2A, indicated by red triangles). Of these mutated sites, two (F239 and R351) involved in IPP substrate-binding in FDS are shown in Figure 2B. The structural feature of FDS is the arrangement of 10 core helices around a large central cavity, and the highly-conserved amino-acids are all located in this cavity [28,29]. These and other conserved FDS features are preserved in CDS, as shown in Figure 2C.

### Selection test

We compared the log likelihood values from different branch models to explore whether the ω ratios varied among different lineages and, particularly, whether the ratios for each ancestral branch to the *FDS1*, *FDS2*, and *CDS* subfamilies differed from those for other branches in the phylogeny. The results are shown in Table 1. The free ratio model (Mf) fit the data significantly better than the one ratio model (M0) (2ΔL = 236.66, P < 0.001), suggesting that the ω ratios varied among lineages (ranging from 0 to 0.941). However, the ω <1 under Mf indicated purifying selection in the gene family. The two ratio models M2a and M2b, which assigned one ω to the ancestral lineages of *FDS1* or *FDS2* and the other ratio ω_0_ to all other branches, produced log likelihood values very similar to the one ratio model and were not significantly better than the one ratio model (for M2a *vs.* M0, 2ΔL = 0.02, P >0.05; for M2b *vs.* M0, 2ΔL = 0.18, P >0.05). In contrast, the two ratio model M2c, with ω_c_ = 0.951 for the lineage ancestral to *CDS* and ω_0_ = 0.122 for all other branches, was significantly better than the one ratio model (2ΔL = 34.22, P <0.001), indicating a significant increase in the ω ratio for the ancestral branch for *CDS.* Finally, the three ratio models, M3a, M3b, and M3c, and the four-ratio model M4a were rejected in favor of M2c (Table 1).

**Table 1:** Parameter estimates under branch, site and branch-site models

**Model**	***p***	**InL**	**Estimate of parameters**	**Models compared**	**2ΔL**
**Branch models**					
M0: ω_0_ = ω_1_ = ω_2_ = ω_c_	1	−10227.98	ω_0_ = ω_1_ = ω_2_ = ω_c_ = 0.130		
Mf: ω free	51	−10109.65	ω: 0 ~ 0.941	Mf *vs.* M0	236.66***
M2a: ω_0_ = ω_2_ = ω_c_, ω_1_	2	−10227.97	ω_0_ = ω_2_ = ω_c_ =0.130, ω_1_ = 0.150	M2a *vs.* M0	0.02
M2b: ω_0_ =ω_1_ = ω_c_, ω_2_	2	−10227.89	ω_0_ = ω_1_ = ω_c_ = 0.129, ω_2_ = 0.190	M2b *vs.* M0	0.18
M2c: ω_0_ = ω_1_ = ω_2_, ω_c_	2	−10210.87	ω_0_ = ω_1_ = ω_2_ = 0.122, ω_c_ = 0.951	M2c *vs.* M0	34.22***
M3a: ω_0_ = ω_c_, ω_1_, ω_2_	3	−10227.88	ω_0_ = ω_c_ = 0.129, ω_1_ = 0.149, ω_2_ = 0.190	M3a *vs.* M2a	0.18
M3b: ω_0_ = ω_2_, ω_1_, ω_c_	3	−10210.85	ω_0_ = ω_2_ = 0.122, ω_1_ = 0.153, ω_2_ = 0.949	M3b *vs.* M2c	0.04
M3c: ω_0_ = ω_1_, ω_2_, ω_c_	3	−10212.06	ω_0_ = ω_1_ = 0.121, ω_2_ = 1.020, ω_c_ = 0.756	M3c *vs.* M2c	−2.38
M4a: ω_0_, ω_1_, ω_2_, ω_c_	4	−10210.82	ω_0_ = 0.122, ω_1_ = 0.153, ω_2_ = 0.148, ω_c_ = 0.942	M4a *vs.* M2c	0.10
**Site-specific models**					
M1: nearly neutral	1	−10152.15	P_0_ = 0.929, P_1_ = 0.071 ω_O_ =0.109, ω_1_ = 1.000		
M2: PositiveSelection	3	−10152.15	P_0_ = 0.929, P_1_ = 0.071, P_2_ = 0.000 ω_0_ = 0.109, ω_1_ = 1.000, ω_2_ =30.688	M2 *vs.* M1	0
M3: discrete (k=2)	5	−10064.21	P_0_ = 0.534, P_1_ = 0.417, P_2_ = 0.048 ω_O_ = 0.028, ω_1_ = 0.223, ω_2_ = 0.670	M3 *vs.* M0	327.54***
M7: beta	2	−10064.80	P = 0.580 q = 3.405		
M8: beta & ω	4	−10061.64	p_0_ = 0.989, p = 0.646, q = 4.203 (p_1_ = 0.011) ω = 1.054	M8 *vs.* M7	6.32*
**Branch-site model**					
model A	3	−10130.51	P_0_ = 0.353, p_1_ = 0.026, p_2_ = 0.621, ω_2_ = 1.442	MA *vs.*M1	43.28***

* significant at p <0.05 level, *** significant at p <0.001 level.

*p*, the number of free parameters for the ω ratio.

The LRTs for M2 *vs.* M1 (2ΔL = 0, P >0.05) suggested that the positive selection model (M2) was not significantly better than the nearly neutral model (M1). Although models M3 and M8 fit the data significantly better than the null models M0 and M7 (for M3 *vs.* M0, 2ΔL = 327.54, P < 0.001; for M8 *vs.* M7, 2ΔL = 6.32, P <0.05), they did not identify sites with an ω value significantly greater than 1.

Given that the branch ancestral to *CDS* exhibited an increased ω (ω_c_ = 0.951) and *CDS* was endowed with a new function in the biosynthesis of terpenoids, branch-site model A was further used to test for evidence of positive selection on the branch ancestral to *CDS* (Table 1). LRTs showed that this model was significantly better than the nearly neutral model M1. The parameter estimates under branch-site model A suggested that 12.6% of codons along the *CDS* branch had been under positive selection, with ω = 1.442. Bayes Empirical Bayes (BEB) analyses showed that at the P >50% level, branch-site model A identified 43 sites as being potentially subjected to positive selection on the CDS branch. At the P >80% level, the following 29 sites were identified: 102M, 103V, 113Q, 166E, 191Q, 198H, 211I, 218T, 220C, 225Q, 235L, 236N, 241Q, 273I, 279M, 281Y, 285N, 290T, 293D, 295D, 300T, 306E, 307C, 334I, 353K, 355A, 356Y, 386C and 394H (amino-acids refer to I13995) (Figure 2A & C). At the P >95% level, three sites, 103V, 218T and 290T, were identified.

## Discussion

Gene duplication is considered to be a major mechanism in the generation of evolutionary novelty and adaptation [23,57,58]. In plants, gene duplication followed by functional divergence is particularly important for the diversification of biochemical metabolites [9,36,37,59]. Isoprenoids are a large and diverse class of metabolites [60] derived from five-carbon isoprene units, which can be classified into regular and irregular forms depending on the bond between isoprene units or monoterpenes, sesquiterpenes and diterpenes according to the number of isoprene units [1,16,61]. FDSs are involved in the biosynthesis of sesquiterpenes, and are encoded by a small gene family. It seems that this gene family has experienced lineage-specific gene expansions multiple times. For example, the two copies of *Arabidopsis thaliana* formed a species-specific clade (Additional file 3). *FDS* copies from *Oryza sative* and *Sorghum bicolor* formed a Poaceae-specific clade (Additional file 3). Based on phylogenetic analyses (Figure 1B), we clearly showed that two rounds of gene duplication occurred in the evolutionary history of the Asteraceae *FDS* gene family. The first round of duplication appears to have occurred in the common ancestor of the Asteraceae, since the genes from Asteraceae formed a monophyletic group separated from the clusters for species from other families (Figure 1B and Additional file 3), and even from the species of two families closely related to Asteraceae (*Nymphoides peltata* of Menyanthaceae and *Platycodon grandiflorus* of Campanulaceae; The Compositae Genome Project, personal communication). The FDS gene duplications in Asteraceae might contribute to the diversity of their sesquiterpenes because of the role of FDSs in the biosynthesis of sesquiterpenes. This is consistent with the large number of sesquiterpenes that have been extracted from Asteraceae [62,63].

The second duplication, which generated the lineage of *CDS*, occurred after the divergence of the Mutisieae from the other tribes of Asteraceae and before the divergence of the tribe Anthemideae. The evidence includes 1) one *FDS* copy in *G. anandria* (Mutisieae) clustered with *FDS1*, while the other clustered with the ancestor of *FDS2* and *CDS*; 2) all of the sampled Anthemideae species had three *FDS* copies (*FDS1*, *FDS2*, *CDS*); and 3) CDS was also cloned from *Aster ageratoides* (tribe Astereae) and *Helianthus annuus* (tribe Heliantheae) that are close relatives of the tribe Anthemideae [39,41]. After the origin of CDS, it developed a new function, involved in the biosynthetic pathway of irregular monoterpenes. The CDS gene is common in the tribe Anthemideae, which is consistent with the fact that its products are typically found in Anthemideae species. Our results suggested that the duplication and divergence of FDS genes has played a major role in determining the novelty of irregular monoterpenes in Anthemideae.

After gene duplication, *CDS* accumulated amino-acid changes toward the change of a substrate. CDSs have four substitutions in the substrate-binding and catalysis sites of FDS: T201 **→** G244 (or S244), F239 **→** Y281, D243 **→** N285, and R351 **→** G393. Substitutions can be divided into either radical or conservative, based on the biochemical properties of the amino-acids [64-67]. For example, substitutions associated with a change of polarity group are defined as radical and those with the polarity group unaltered as conservative [65,67]. In the present study, except for T201 **→** S244, all the substitutions are radical, which is consistent with the fact that the evolution of new function requires alterations in the biochemical properties of the amino-acid sequence [67]. F239 and R351 in FDS are involved in binding of the nucleophilic substrate IPP [30,31]. The radical replacements of these two sites in *CDS* are in good agreement with the finding that *CDS* does not prefer IPP as a nucleophilic reagent. F239 in FDS binds IPP through hydrophobic interactions [30,31]. The corresponding residue is Y281 in CDS, which, owing to the polarity of the hydroxyl, may not interact with IPP through hydrophobic interactions. R351 in FDS interacts with the pyrophosphate moiety of IPP through water-mediated hydrogen bonds [30,31] (Figure 2B). The radical replacement of R351 **→** G393 involves changes in the charge (R is positively-charged and G is nonpolar) and the molecular volume of the amino-acids (R has a larger side-chain than G), which could affect the IPP binding. Hence, these two substitutions might explain why *CDS* does not prefer IPP as a substrate.

Substitutions can change the function of duplicated genes, and may be due to either a relaxation of purifying selection or to the action of positive selection [19,23,32,33]. Branch-site model A provided evidence of positive selection acting at 29 (p >80) sites along the branch ancestral to *CDS* (Figure 2A). Interestingly, Y281 (F239 in FDS) and N285 (D243 in FDS) noted above were found to be under positive selection by the branch-site model, suggesting the important role of positive selection in the functional evolution of CDS. The biochemical context of substitutions that were under positive selection is consistent with a scenario involving the adaptive evolution of CDS. These sites (p>80%) were scattered throughout the primary sequences (Figure 2A), whereas in the three-dimensional structures (Figure 2C), they clustered in the large central cavity. Among these sites, two (102M and 103V) were located in conserved region I, and nine (279M, 281Y, 285N, 290T, 293D, 295D, 300T, 306E and 307C) were located in conserved region V. They are all conserved in the *FDS* gene family and important for the precise function of the protein [27-31]. The mutations at these sites suggested that their importance for the enzymatic activity of FDS was altered in CDS. A few sites that were detected to have experienced positive selection with high probability may be responsible for the novel function of CDS. Further studies using site-directed mutagenesis are needed to determine whether these positively-selected sites, especially those with high posterior probability (103V, 218T and 290T), confer an ability on CDS to discriminate different substrate types.

It has been proposed that positive selection promotes the functional divergence of gene family members encoding enzymes involved in secondary metabolism because secondary products are considered to be a response to challenges imposed by the environment [36,37]. For example, the methylthioalkylmalate synthase gene (*MAM*) controls an early step in the biosynthesis of glucosinolates, which play an important role in *Arabidopsis thaliana* and other crucifers’ defense against herbivorous insects [37]. Benderoth et al. [37] found that positive selection had driven the evolution of *MAM2* that originated from a lineage-specific duplication of *MAMa* in *A. thaliana*. Another example is the SABATH gene family of methyltransferases, which encodes enzymes catalyzing the formation of a variety of secondary metabolites in plants such as those that contribute to floral scent and plant defense. Branch-site analysis suggested that positive selection for a single amino-acid change promoted the substrate discrimination of salicylic acid methyltransferase [68]. Here, We provide an additional example in which positive selection has promoted the functional divergence of duplicated genes in a secondary metabolic pathway. The adaptive evolution of the CDS gene at the molecule level is consistent with the adaptive roles of the products of CDS (irregular monoterpenes), and plays an important role in plant survival.

Many models have been proposed to explain the evolutionary fates of duplicated genes, including neofunctionalization, duplication-degeneration-complementation (or subfunctionalization) and escape from adaptive conflict (EAC) models [18-25]. Compared with CDS gene in which adaptive selection has been detected, FDS2 gene, as a sister duplicated copy of *CDS*, did not show any signature of positive selection. So, it seems that the evolution of *FDS2* and *CDS* are not consistent with the predictions of the EAC model, where both duplicated copies would evolve under positive selection [24,25]. Particularly, CDS has a ~ 50-amino acid extension at its N-terminal, which was identified as a plastidial transit peptide, in agreement with the Category II-c model (Gene duplication with a modified function) [24]. However, the peptide of CDS shares little similarity with any other sequence database entries by Blast searches. Further work including functional analysis and the exploration on the origin of the peptide of CDS would provide insights into the evolutionary fate of the *FDS* gene family in Asteraceae.

## Conclusions

Based on phylogenetic analyses of *FDS* sequences, we demonstrated that two duplication events occurred in the evolution of the Asteraceae *FDS* gene family. The first occurred in the common ancestor of the Asteraceae and the second after the divergence of the Mutisioideae from the other tribes, but before the birth of the Anthemideae tribe. We found that CDS accumulated four mutations in sites homologous to the substrate-binding and catalysis sites of FDS: T201 **→** G244 in conserved region IV, D243 **→** N285 in the first aspartate in conserved region V, F239 **→** Y281 in region V, and R351 **→** G393 in the C-terminal. Of the four replaced sites of FDS, F239 and R351 are involved in the binding of the nucleophilic substrate isopentenyl diphosphate. Likelihood analyses of a branch-site model provided evidence of positive selection acting on 29 sites (p >80) and 3 sites (p >95) on the branch ancestral to *CDS*. Positive selection associated with gene duplication has played a major role in the evolution of *CDS*.

## Abbreviations

BI: Bayesian inference; CDS: Chrysanthemyl diphosphate synthase; DMAPP: Dimethylallyl diphosphate; EAC: Escape from adaptive conflict; EST: Expressed sequence tags; FDS: Farnesyl diphosphate synthase; IPP: Isopentenyl diphosphate; MAM: Methylthioalkylmalate synthase; ML: Maximum likelihood; RACE: Rapid amplification of cDNA ends.

## Competing interests

The authors declare that they have no competing interests.

## Authors’ contributions

GYR, YPG and PLL designed the experiments. PLL and JNW conducted the experiments. GYR, YPG, SG and PLL analyzed and interpreted the data. GYR, YPG, SG and PLL wrote the manuscript. All authors read and approved the final manuscript.

## Supplementary Material

Additional file 1 Primers for RACE. Click here for file

Additional file 2 Sequences included in this study. Click here for file

Additional file 3 **Maximum likelihood tree of FDS gene family from different plants.** Numbers next to branches are bootstrap percentages from Maximum Likelihood analysis, and posterior probabilities from Bayesian analysis. Click here for file
