# Supplementary material for: Adaptive evolution of the chrysanthemyl diphosphate synthase gene involved in irregular monoterpene metabolism
Source: BMC Evol Biol. 2012 Nov 8;12:214. doi: 10.1186/1471-2148-12-214 (PMC3518182; doi:10.1186/1471-2148-12-214)
Supplement: Additional file 1 — Primers for RACE. [file 1471-2148-12-214-S1.pdf]

Primers for RACE

primers	Sequence (5'-3')	
3'-RACE-18	GCTGTCAACGATACGCTACGTAACGGCATGACAGTG(T)18	3'-RACE RT
GSP2-nested	CGCTACGTAACGGCATGACAGTG	3'-RACE PCR
AP	GGCCACGCGTCGACTAGTAC(T)17	3'-RACE RT
AUAP	GGCCACGCGTCGACTAGTAC	3'-RACE PCR
AAP	GGCCACGCGTCGACTAGTACGGGIIGGGIIGGGIIG	5'-RACE 1st PCR
AUAP	GGCCACGCGTCGACTAGTAC	5'-RACE 2nd PCR
asi_fds1F1	TCCGAGGAAAGCCTTACTATGTG	3'-RACE 1ST PCR for Asi_FDS1
asi_fds1F2	CGGACAGATGATTGATTTGATCACT	3'-RACE 1st PCR for Asi_FDS1
asi_fds1r2	TCTCCAACTAGTGTAGTGATCAA	5'-RACE RT for Asi_FDS1
asi_nfds2r2	AACCTCGTTGAACAGGTCCAC	5'-RACE 1st PCR for Asi_FDS1
asi_nfds2r3	TCCACATAGTAAGGCTTTCCTC	5'-RACE 2nd PCR for Asi_FDS1
asi_fds2F1	TTATTACGCTGATCTACTAGACCTC	3'-RACE 1st PCR for Asi_FDS2
asi_fds2F2	GCTCTCGAAGTATTCTTTGTCTAC	3'-RACE 2nd PCR for Asi_FDS2
asi_nfds1r1	TGAACTGAACAATCCTCC	5'-RACE RT for Asi_FDS2
asi_nfds1r2	CTGTCCAACAAGTGTTGTGATT	5'-RACE 1st PCR for Asi_FDS2
asi_nfds1r3	TCATTGAAGAGGTCTAGTAGATCAG	5'-RACE 2nd PCR for Asi_FDS2
asi_fds5cF1	GATTGATGAGTTAGCTAGACTAGC	3'-RACE 1st PCR for Asi_FDS5
asi_fds5f2	TTAACCGTCGGATTGTTGAGT	3'-RACE 2nd PCR for Asi_FDS5
asi_nfds5r1	ACTGTTTGAAATTCGGTC	5'-RACE RT for Asi_FDS5
asi_nfds5r2	GTCTCATTGAAGAGGCCGAG	5'-RACE 1st PCR for Asi_FDS5
asi_nfds5r3	CCGAGAAGATGAACGTAATAAGTC	5'-RACE 2nd PCR for Asi_FDS5
lav_fds1F2	TTGATCACTACACTTGTCGGAG	3'-RACE 1st PCR for Lav_FDS1
lav_fds1F3	CTCAAAGTATTCATTGTCCATTCAC	3'-RACE 2nd PCR for Lav_FDS1
fds1-fan-1	CTCTCCGACAAGTGTAGTG-3'	5'-RACE RTfor Lav_FDS1
fds1-fan-2	CGTTGAAAAGGTCCACAAGATCC	5'-RACE 1st PCR for Lav_FDS1
fds1-fan-3	AGATCCACATAGTAAGGCTTTC	5'-RACE 2nd PCR for Lav_FDS1
lav_fds2F1	ATTATGCTGATCTACTAGACCTC	3'-RACE 1st PCR for Lav_FDS2
lav_fds2F2	GCTCTCGAAGTATTCTCTGTCTAC	3'-RACE 2nd PCR for Lav_FDS2
fds2-fan-1	TGTCCAACAAGTGTCGTG-3	5'-RACE RT for Lav_FDS2
fds2-fan-2	CGTTGAAGAGGTCTAGTAGATCAG	5'-RACE 1st PCR for Lav_FDS2
fds2-fan-3	CAGCATAATAAGCCTCCCCTC	5'-RACE 2nd PCR for Lav_FDS2
lav_fds5F1	GGCTTATTATGTGCATCTAGTG	3'-RACE 1st PCR for Lav_FDS5
lav_fds5F2	GTTATTGCTACACTTGATGGGA	3'-RACE 2nd PCR for Lav_FDS5
fds5-fan-1	CCATCAAGTGTAGCAATAAC	5'-RACE RT for Lav_FDS5
fds5-fan-2	TCCACAGACTGTTTGAAATTCCG	5'-RACE 1st PCR for Lav_FDS5
fds5-fan-3	CGTTGAATAGATCCACTAGATGC	5'-RACE 2nd PCR for Lav_FDS5
Tco_fds1f1	CCGAGGAAAGCCTTACTATGTGG	3'-RACE PCR for Tco_FDS1
Tco_fds1r1	TCGGCGGTGAACAGACAAT	5'-RACE RT for Tco_FDS1
Tco_fds1r2	ACAGGTCCACAAGATCCACATAGT	5'-RACE 1st PCR for Pco_FDS1
Tco_fds1r3	TAGTAAGGCTTTCCTCGGAAATGA	5'-RACE 2nd PCR for Pco_FDS1
Tco_fds2f1	ATCCCAAGAATCCTAAAGAAACA	3'-RACE 1st PCR for Pco_FDS2
Tco_fds2f2	TTATGCTGATCTACTAGACCTCTTC	3'-RACE 2nd PCR for Pco_FDS2
Tco_fds2r1	CCGCCGGTGAGTAGACAAG	5'-RACE RT for Pco_FDS2
Tco_fds2r2	TGAAGAGGTCTAGTAGATCAGCAT	5'-RACE PCR for Pco_FDS2
Tco_fds5f1	CCTCTTCAATGAGACCGAATTT	3'-RACE 1st PCR for Pco_FDS5
Tco_fds5f2	TTCTATGACTCTTAACCGTCGGA	3'-RACE 2nd PCR for Pco_FDS5
Tco_fds5r1	TCCGACGGTTAAGAGTCATA	5'-RACE RT for Pco_FDS5
Tco_fds5r2	TGAAATTCGGTCTCATTGAAGAG	5'-RACE PCR for Pco_FDS5
Lvu_1f1	GAGGAAAGCCTTACTATGTGGAT	3'-RACE 1st PCR for Lvu_FDS1
Lvu_1f2	AGTATTCATTGTCTGTTCACCGCC	3'-RACE 2nd PCR for Lvu_FDS1
Lvu_1fr1	GGCGGTGAACAGACAATGA	5'-RACE RT for Lvu_FDS1
Lvu_1fr2	AAGATCCACATAGTAAGGCTTTCC	5'-RACE 1st PCR for Lvu_FDS1
Lvu_1fr3	GAATTCTCGGGACATGGTTGCGA	5'-RACE 2nd PCR for Lvu_FDS1
Lvu_2f1	TAAATGATGGAGTTGTTCTCCGC	3'-RACE 1st PCR for Lvu_FDS2
Lvu_2f2	CCATATCCCAAGAATCCTAAAGA	3'-RACE 2nd PCR for Lvu_FDS2
Lvu_2fr1	AAGAGGTCTAGTAGATCAGCATAA	5'-RACE RT for Lvu_FDS2
Lvu_2fr2	AATAAGCCTTCCCTCGGAAATGT	5'-RACE 1st PCR for Lvu_FDS2
Lvu_2fr3	TGTTTCTTTAGGATTCTTGGGAT	5'-RACE 2nd PCR for Lvu_FDS2
Aster_fds12f1	AGTATTCATTGTCTATTCACCGC	3'-RACE 1st PCR for Ast_FDS1
Aster_fds12f2	CGCCGGATTGTGCAGTACAA	3'-RACE 2nd PCR for Ast_FDS1
aster_2f1	GTATTCTTTGTCGATCCACCGT	3'-RACE 2nd PCR for Ast_FDS2
Aster_fds12r1	CAATCCGGCGGTGAATAGA	5'-RACE RT for Ast_FDS1
Aster_fds12r2	CAAGTGTTGTGATCAAATCAATC	5'-RACE 1st PCR for Ast_FDS1
Aster_fds12r3	CATTGAATAGGTCCACTAAATCC	5'-RACE 2nd PCR for Ast_FDS1
aster_2r2	TCCGACGGTGGATCGACAAAGA	5'-RACE RT for Ast_FDS2
aster_2r3	TCATTGAAGAGGTCCAGAAGAT	5'-RACE 1st PCR for Ast_FDS2
Aster_fds2r4	GAGGTCCAGAAGATCCACATAG	5'-RACE 2nd PCR for Ast_FDS2
Aster_fds5r1	TATGCAACGATGCGACGGT	5'-RACE RT for Ast_FDS5
Aster_fds5r2	TTCCAACATGTGTAGCAATAGCA	5'-RACE 1st PCR for Ast_FDS5
Aster_fds5r3	CATTGAAGAGGTCCAGTAGATG	5'-RACE 2nd PCR for Ast_FDS5
tara_1F1	AGAGGAAAGCCGTATTACATTG	3'-RACE 1st PCR for Tar_FDS1
tara_1f2	TTCATTGTCCATTCACCGCAGG	3'-RACE 2nd PCR for Tar _FDS1
tara_5f2	ACTCGATCTCTTCAATGAGGTGG	3'-RACE 1st PCR for Tar _FDS2
tara_5f3	TATCTATTCATCGCCGGATTGTT	3'-RACE 2nd PCR for Tar _FDS2
tara_1r3	GAAGAATTATTCCATCATTTGCA	3'-RACE 2nd PCR for Tar _FDS5
tara_5r1	TCCGGCGATGAATAGATAAAG	5'-RACE RT for Tar _FDS1
tara_5r2	TTGAAGAGATCGAGTAGGTCCAC	5'-RACE 1st PCR for Tar _FDS1
tara_5r3	ATATGGTTGCGAAGAACAACTCC	5'-RACE 2nd PCR for Tar _FDS1
ha1_FDSII	GTGCTTGATGATATCATGGA	3'-RACE 1st PCR for Ha_FDS1
ha_1f2	AAGGTTGGTATGATTGCTGC	3'-RACE 2nd PCR for Ha _FDS1
ha_1r1	TCTTGGGATATGGTTACGAA	5'-RACE RT for Ha _FDS1

ha_1r1	TCTTGGGATATGGTTACGAA	5'-RACE 1st PCR for Ha _FDS1
ha_1r2	GCAGCAATCATACCAACCTT	5'-RACE 2nd PCR for Ha _FDS1
ha_5f1	ATCGATCTAAAGTCCAAATTCTTG	3'-RACE 1st PCR for Ha _FDS5
ha_5rf2	GGTTCAGAATACCAGAGGTTAA	3'-RACE 2nd PCR for Ha _FDS5
ha_5rr1	TTAGGATTCGACGGACATG	5'-RACE RT for Ha _FDS5
ha_5rr2	ATGGTTGGAAAGAAGAGCACC	5'-RACE 1st PCR for Ha _FDS5
ha_5rr3	CAGCAATCATATTAACCTCTGG	5'-RACE 2nd PCR for Ha _FDS5
Gerb_12f1	GAGGTAGAGTTCCAAACAGCAT	3‘-RACE 1st PCR for Ge_FDSb
Gerb_12f2	ATTCTCTGTCTATTCACCGCCG	3‘-RACE 2nd PCR for Ge_FDSb
Ger_1r1	GGCAACTGGAAGGTAAAATG	5‘-RACE RT for Ge_FDSb
Ger_1r2	TCTCCAACAAGTGTGGTGATC	5‘-RACE 1st PCR for Ge_FDSb
Ger_1r3	CTCGGAAATGTTTCTTTAGGAT	5‘-RACE 2nd PCR for Ge_FDSb
Ger_XF1	GATTGCCGCAAATGATGCAGTAG	3‘-RACE 1st PCR for Ge_FDS1
Ger_XF2	CCCTCGAAGTATTCGTTGTCTG	3‘-RACE 2st PCR for Ge_FDS1
Ger_XR1	CCTGTGCACTAGTTATGTTTGG	5‘-RACE RT for Ge_FDS1
Ger_XR2	CCCTCGAAGTATTCGTTGTCTG	5‘-RACE 1st PCR for Ge_FDS1
Ger_XR3	GTTGATTGATTTGATCACTACAA	5‘-RACE 2nd PCR for Ge_FDS1

Asi: *A. asiatica*, Lav: *C. lavdulifolium*, Pco: *P. coccineum*, Lvu: *L. vulgare*,
Ast: *A. ageratoides*, Tar: *T. mongolicum*, Ha: *H. annus*, Ge*: G. Anandria.*
