# Supplementary material for: Adaptive evolution of the chrysanthemyl diphosphate synthase gene involved in irregular monoterpene metabolism
Source: BMC Evol Biol. 2012 Nov 8;12:214. doi: 10.1186/1471-2148-12-214 (PMC3518182; doi:10.1186/1471-2148-12-214)
Supplement: Additional file 2 — Sequences included in this study. [file 1471-2148-12-214-S2.pdf]

Sequences included in this study

Taxon	Sequences	Assembly information or GenBank Accession Number
Asteraceae		
Asteroideae		
Heliantheae		
Helianthus exilis	FDS1,FDS1b, CDS	EE643694+EE646893+EE647943+EE633807+EE644682+EE648258+EE648132, EE639621, EE633074+EE638653
Helianthus annua	**FDS1, CDS**	**JX424564, JX424565**
Anthemideae		
Achillea asiatica	**FDS1, FDS2, CDS**	**JX424551, JX424552, JX424553**
Chrysanthemum lav	**FDS1, FDS2, CDS**	**JX424554, JX424555, JX424556**
Artemisia tridentate	FDS1, FDS2, CDS	AY308477, AY308476, AY308478
Pyrethrum coccineum	**FDS1, FDS2, CDS**	**JX424559, JX424560, JX424561**
Pyrethrum cin	CDS	I13995
Leucanthemum vulgare	**FDS1, FDS2, CDS**	**JX424557, JX424558**
Astereae		
Aster ageratoides	**FDS1, CDS**	**JX424562, JX424563**
Cichorioideae		
Cichorieae		
Cichorium intybus	FDS1, FDS2, FDS2a	EH694645+EH700642+DT212244, EH690889+DT211478, EH710308
Lactuca saligna	FDS1, FDS2	DW055927+DW069905+DW065409+DW061488+DW066168, DW044506
Taraxacum officinale	FDS1, FDS2	DY817407+DY825488+DY823614+DY823660+DY822452+DY822468+ DY842588+DY842976+ DY838800, DY826955
Taraxacum mongolicum	**FDS1, FDS2**	**JX424566, JX424567**
Carduoideae		
Cardueae		
Centaurea solstitialis	FDS1, FDS2	CNSM13746+CNSM9065+CNSM10077, EH785623+EH774553+EH780969
Carthamus tinctorius	FDS1	EL378519+EL396505+EL395715+EL396505+EL398647+ EL379091+EL510343
Cynara scolymus	FDS1	GE606813+GE601816+GE602983+GE602504+GE612594+GE604066+GE608099+GE593354+GE598785+GE596282
Mutisioideae		
Mutisieae		
Gerbera anandria	**FDS1, FDSb**	**JX424569, JX424568**
Gerbera hybrida	FDS25	AJ755145
Barnadesioideae		
Barnadesia spinosa	FDS1	GE542345+GE526692
Apiaceae		
Centella asiatica	FDS	AY787627
Solanoideae		
Solanum lycopersicum	FDS	AF048747
Solanoideae		
Capsicum annuum	FDS	X84695
Lamiaceae		
Mentha x piperita	FDS	AF384040
Gentianaceae		
Gentiana lutea	FDS	AB017371

Abbriviations: *Chrysanthemum lav*, *Chrysanthemum lavandulifolium; Pyrethrum cin*, *Pyrethrum cinerariaefolium.*Bold represents sequences cloned in this study.
