# Supplementary figures and images for: Adaptive evolution of the chrysanthemyl diphosphate synthase gene involved in irregular monoterpene metabolism
Source: BMC Evol Biol. 2012 Nov 8;12:214. doi: 10.1186/1471-2148-12-214 (PMC3518182; doi:10.1186/1471-2148-12-214)

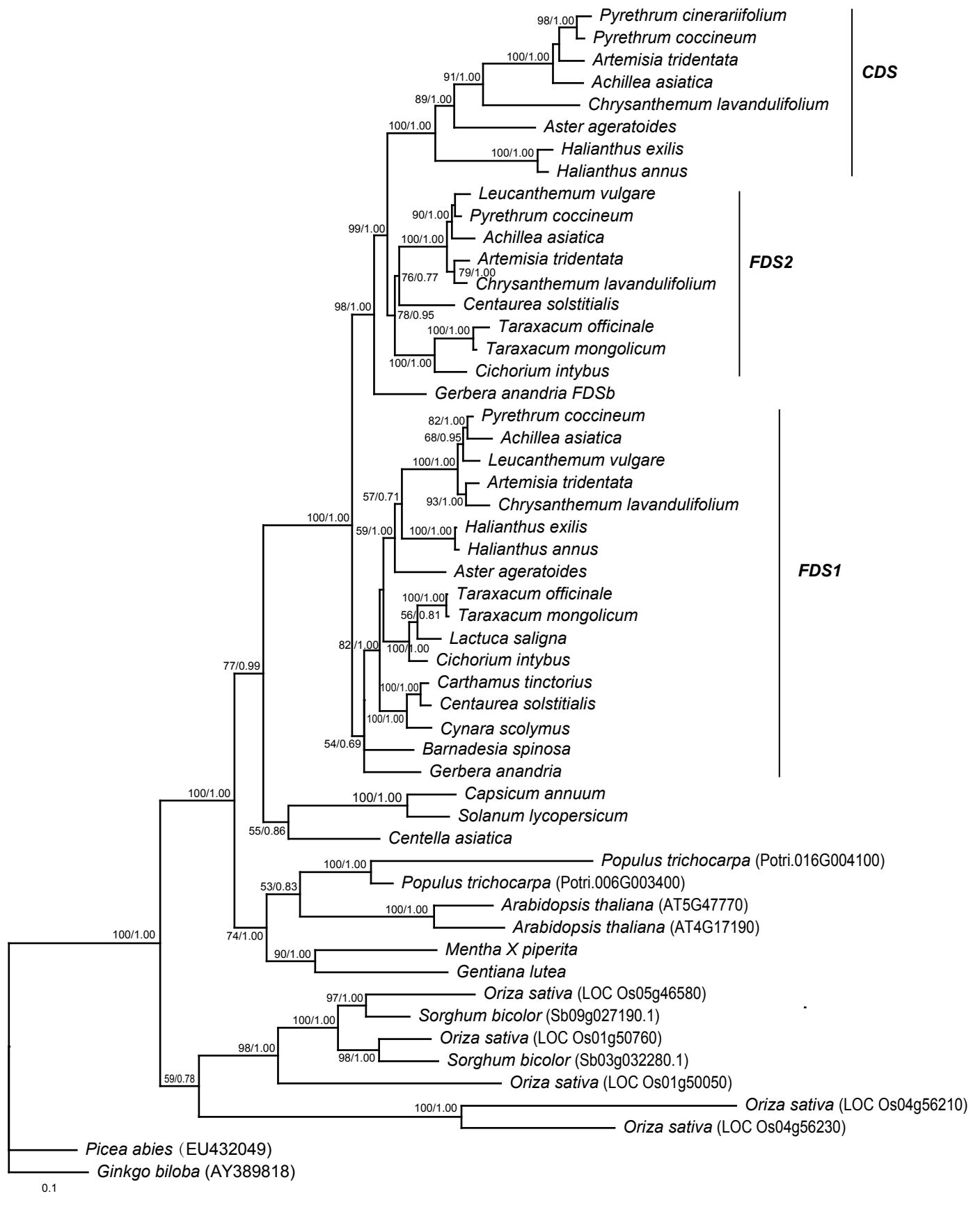

Supplement: Additional file 3 — Maximum likelihood tree of FDS gene family from different plants. Numbers next to branches are bootstrap percentages from Maximum Likelihood analysis, and posterior probabilities from Bayesian analysis. [file 1471-2148-12-214-S3.pdf]
